# Schmallenberg Virus in Calf Born at Term with Porencephaly, Belgium

**DOI:** 10.3201/eid1806.120104

**Published:** 2012-06

**Authors:** Mutien-Marie Garigliany, Bernd Hoffmann, Marc Dive, Arnaud Sartelet, Calixte Bayrou, Dominique Cassart, Martin Beer, Daniel Desmecht

**Affiliations:** University of Liège, Liège, Belgium (M.-M. Garigliany, M. Dive, A. Sartelet, C. Bayrou, D. Cassart, D. Desmecht);; Friedrich-Loeffler-Institut, Greifswald–Insel Riems, Germany (B. Hoffmann, M. Beer)

**Keywords:** Schmallenberg virus, orthobunyavirus, hydranencephaly, viruses, calf, porencephaly, Belgium

**To the Editor:** From the end of August through the end of October 2011, a clinical syndrome involving adult cattle and the fetuses of pregnant cows emerged in the border area between the Netherlands and North Rhine-Westphalia, Germany (*1*). The syndrome was characterized by nonspecific clinical signs (fever, decreased milk production), severe diarrhea, and some abortions. A metagenomic analysis was conducted on pooled samples from cattle with acute signs on a farm in the city of Schmallenberg, Germany.

The analysis detected nucleotide sequences homologous to arthropod-borne Akabane, Aino, and Shamonda viruses, all belonging to the family *Bunyaviridae*, genus *Orthobunyavirus*, and Simbu serogroup (*1*). Real-time PCR detected the genomic RNA of the new and emerging virus, tentatively designated Schmallenberg virus (SBV), in the blood of adult cattle, abdominal fluid of a stillborn calf, and brains of lambs born with birth defects on dozens of farms in the Netherlands, Germany, and Belgium. No data are yet available to predict how the emerging virus might affect the cattle industry. We report the case of a 1-week old calf with severe central nervous system (CNS) lesions probably caused by in utero infection with the new virus.

In Belgium in January 2012, a Belgian Blue multiparous cow gave birth to a 45-kg female calf that was morphologically normal but hypertonic and hyperreflexic. Pregnancy had proceeded uneventfully and lasted 9 months and 4 days. Spontaneous reflexes such as sucking, swallowing, micturition, defecation, and crying were completely preserved, but the calf was unable to stand, and its consciousness alternated from mild to severe depression. It was obviously blind and showed ventrolateral strabismus, but the pupils functioned normally. Muscle tone was permanently increased, as indicated by tetanus-like erection of the ears and by a violent but brief startle response to the slightest acoustic or tactile stimulation (Figure). When the calf was placed upright, loss of conscious proprioception was obvious; it maintained its position only a few seconds before collapsing. Altogether, the clinical signs suggested severe dysfunctions of the cerebral cortex, basal ganglia, and mesencephalon. The calf drank from a bottle twice a day for a week, but then was euthanized for humane reasons (infected decubital ulcers).

**Figure:**
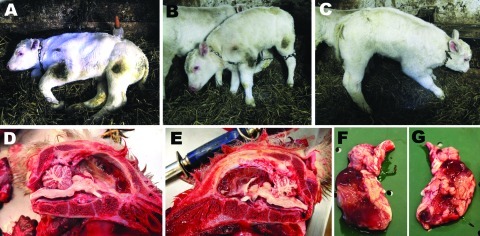
A 7-day old, female, Schmallenberg virus–positive calf showing severe central nervous system dysfunctions (A–C) and lesions (D–E). A) Spontaneously lying down; B–C) standing with assistance; D–G) porcencephaly, either with the encephalon in place (D–E) or extracted (F–G). The cerebral hemispheres were replaced by 2 thin-walled, fluid-filled cysts (diamonds) with some floating islets and peninsulae corresponding to preserved cortex (stars). The cerebrum was variably preserved, the occipital lobes were totally liquefied, and the outer layers of some parts of the temporal and frontal lobes were irregularly preserved. The cerebellum, brainstem, and diencephalon appeared normal in shape and volume.

At necropsy, the cerebellum, brainstem, and diencephalon appeared normal in shape and volume (Figure). However, the cerebral hemispheres were replaced by 2 thin-walled, fluid-filled cysts with some floating islets and peninsulae corresponding to preserved cortex. There was variable preservation of the cerebrum, total liquefaction of occipital lobes, and irregular preservation of the outer layers of some parts of the temporal and frontal lobes. Altogether, the picture was compatible with severe porencephaly or hydranencephaly. The spine showed no sign of scoliosis, and movement of the limb joints was not restricted (i.e., no arthrogryposis).

Samples were removed from the remnants of the cerebrum, diencephalon, and organs (thymus, lung, myocardium, jejunum, ileum, mesenteric lymph node, liver, spleen, kidney, and striated muscle), and 3 independent real-time PCR protocols were conducted to detect genomes of bovine viral diarrhea/mucosal disease virus, bluetongue virus serotype 8, and the novel SBV. Initial retrotranscription of the RNA genomes was followed by quantitative (real-time) PCR. The process was conducted by using our procedures (*2*) and, for SBV, by following the protocol and using recently developed control reagents as described (*1*). The SBV genome was detected in only CNS samples (quantification cycle value 28.8); bovine viral diarrhea/mucosal disease virus and BTV-8 genomes were not detected. The new virus genome load was 1.61 × 10^4^ copies per gram of cerebrum sample.

Taken together, the above data suggest that, like other Simbu serogroup viruses, the new virus crosses the placenta, contaminates the bovine fetus, infects the fetus’ CNS, and causes necrosis and/or developmental arrest of the cerebral cortex. Unlike the viruses mentioned above (*3**,**4*), and provided this case is not an exception, the SBV genome seems to persist in the infected fetus and is detectable after birth by real-time reverse transcription PCR, despite gestation length. Although reliable reagents for detecting seroconversion are temporarily unavailable, the persistence of the new virus in fetal tissue should greatly facilitate the epidemiologic monitoring of the emergence and spread of the new virus.

When calves from experimentally infected dams are infected with the closest phylogenetic relative to SBV, Akabane virus, porencephaly develops during gestational days 62–96 (*5*). If the same is true for the new virus, the above calf was probably infected during June 9–July 13, 2011. Therefore, it is hypothesized that infected arthropods were already circulating in the village of Hamois-in-Condroz (50°24′56′′N, 5°8′7′′E), which is ≈240 km southwest of Schmallenberg (51°8′42′′N, 8°17′18′′E), ≈2 months before the emergence of the clinical syndrome that led to the identification of the new virus.
